# Can molecular cell biology explain chromosome motions?

**DOI:** 10.1186/1742-4682-8-15

**Published:** 2011-05-27

**Authors:** Daniel H Shain, L John Gagliardi

**Affiliations:** 1 Department of Biology, Rutgers The State University of New Jersey, 315 Penn St., Camden, NJ 08102, USA; 2 Department of Physics, Rutgers The State University of New Jersey, 315 Penn St., Camden, NJ 08102, USA

## Abstract

**Background:**

Mitotic chromosome motions have recently been correlated with electrostatic forces, but a lingering "molecular cell biology" paradigm persists, proposing binding and release proteins or molecular geometries for force generation.

**Results:**

Pole-facing kinetochore plates manifest positive charges and interact with negatively charged microtubule ends providing the motive force for poleward chromosome motions by classical electrostatics. This conceptual scheme explains dynamic tracking/coupling of kinetochores to microtubules and the simultaneous depolymerization of kinetochore microtubules as poleward force is generated.

**Conclusion:**

We question here why cells would prefer complex molecular mechanisms to move chromosomes when direct electrostatic interactions between known bound charge distributions can accomplish the same task much more simply.

## Introduction

Molecular mechanisms underlying mitosis, particularly those associated with directed chromosome movement during the cell cycle, have been pursued intensely over the past two decades with no clear picture emerging--or is there? Recent experiments identify positively charged kinetochore-associated molecules (e.g., Ndc80/Hec1) that likely interact with negatively charged microtubule ends to generate electrostatic-dependent poleward forces that drive chromosome motion [1,2]. This concept diverges from the conventional "molecular cell biology" paradigm, but does not stray far from molecular-based approaches that require specific binding proteins or molecular geometries for force generation. In fact, considerable time and resources are being invested pursuing molecular machinery that may not exist.

## Discussion

**Figure 1:**
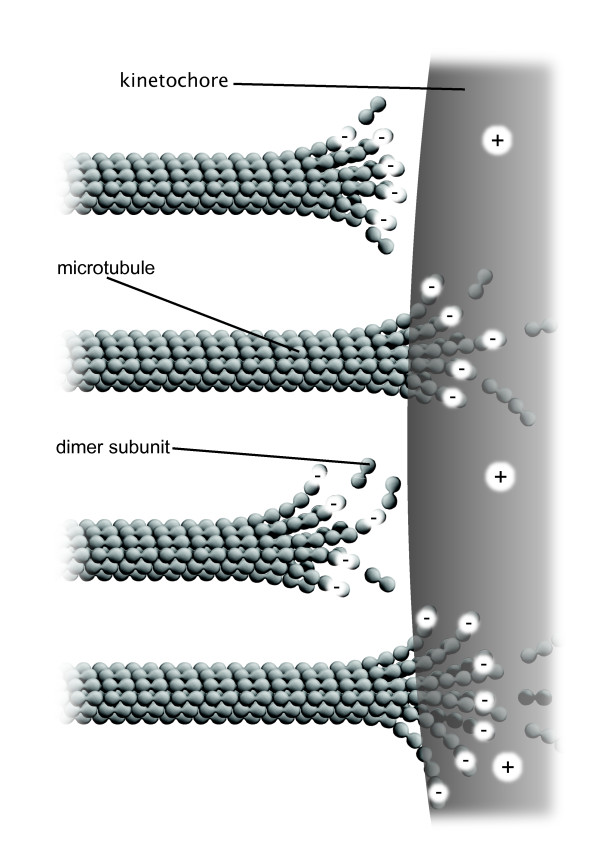
**Nanoscale electrostatic disassembly force at a charged kinetochore**. A poleward force results from an electrostatic attraction between negatively charged microtubule free ends and an oppositely charged kinetochore. A few of the numerous microtubules that attach to each kinetochore are shown.

Indeed, current thought on mitotic motions is shifting from a molecular to a more electrostatics-based framework [1-3], and perhaps not too surprisingly in light of theoretical predictions made almost a decade ago, which have gone mostly unrecognized [4-6]. Specifically, pole-facing kinetochore plates manifest positive charges and interact with negatively charged microtubule ends providing the motive force for poleward chromosome motions (Figure 1). This conceptual scheme explains dynamic tracking/coupling of kinetochores to microtubules and the simultaneous depolymerization of kinetochore microtubules as poleward force is generated. Charges, of course, are on the molecules (i.e., microtubules, kinetochore binding proteins), but the molecules are mere carriers of charges that cause chromosome motions by classical electrostatics. Note that antipoleward chromosome motions are also integrated into the complex motions of mitosis [4-6]. Collectively, this concept is very different from the electrostatics-based, molecular binding and release mechanisms presently suggested--but not elucidated--in recent literature [1,2]. For example, a lock and key mechanism involving the calponin homology domain, which has recently been associated with kinetochore attachments to microtubule ends [7,8], does not explain complex chromosome motions during prometaphase, metaphase and anaphase. Alternatively, we suggest that calponin may serve to position and stabilize the microtubule-kinetochore end-on attachment, while the highly positive, unstructured tail of Ndc80/Hec1 is likely the dynamic electrostatic link with microtubule ends.

Perhaps the most surprising part of this story is the untimely resistance to classical electrostatics by the cell biology community. For example, critiques including "...groundless speculations in which the authors [*sic*] attempted to explain chromosome motions by nanoscale electrostatics and unnecessary sophistry..." [9], and requiring "...hypothetical long-range electrostatic forces..." [10] suggest an inherent bias against--and general unawareness of--electrostatic forces and their fundamental role in cellular processes. In response, nanoscale electrostatics has in fact emerged as a primary focus for chromosome movements [1,2], and is far from hypothetical in light of water layering [11] and reduction of the dielectric constant between charged protein surfaces [12].

To gain perspective on this subject, it may be instructive to consider the problem of cell division in an evolutionary context, and more specifically in an ancestral cell that lacked "modern" molecular machinery. Clearly, cells have been dividing since the origin of life, and the mechanisms underlying this fundamental process in modern cells are likely derived from some ancestral state--just like other cellular processes (e.g., translation, splicing) were likely derived from ancestral, catalytic RNAs that were later supplemented with supporting proteins. In a simple cell, all chromosome movements during mitosis are readily explained by electrostatic interactions between core components of the system (i.e., charged DNA, microtubules), without the requirement for supplemental protein machinery [4-6]. Why then should modern cells be expected to conduct mitosis in a fundamentally different way (i.e., the molecular cell biology paradigm)? Rather, a more parsimonious view might consider mitosis as an emergent property, with specialized DNA and microtubules as key players and electrostatics as the driving force. Analogous with other cellular processes, supplemental protein machinery likely arrived later to increase efficiency in an increasingly complex cellular environment.

Our current bottleneck in understanding mitotic chromosome movements seems reminiscent of another challenging question in our imperfect scientific history, namely the self-imposed constraints of ancient Greek astronomers in trying to explain geocentric planetary motions with perfect circles. Indeed, layers of epicycles were incorporated into an increasingly complex scheme of integrated circles that was "understood" by only the best natural philosophers of the time. It took ~2,000 years of scientific work by Brahe, Galileo, Kepler and Newton to achieve the simplicity of a modern theory based on a different conceptual scheme, i.e., elliptical orbits in a heliocentric solar system.

## Conclusions

Twenty years ago, Guenter Albrecht-Buehler lamented the view of many cell biologists that "molecular analysis of cellular functions" is the only acceptable approach to cell biology [13], yet this precarious ideology seems even more entrenched in current cell science. Imposing molecular approaches (e.g., binding and release mechanisms) at the outset does not preserve scientific open-mindedness in solving nature's riddles. Although much good science has been done in molecular biology, do we really want modern cell biologists spiraling around epicycles like ancient Greek astronomers? Instead, perhaps we should ask why cells would prefer complex molecular mechanisms to move chromosomes when direct electrostatic interactions between known bound charge distributions can accomplish the same task much more simply.

## Competing interests

The authors declare that they have no competing interests.

## Authors' contributions

DHS made intellectual contributions and drafted the manuscript. LJG conceived the study. All authors read and approved the final manuscript.
